# Defects in the Neuroendocrine Axis Contribute to Global Development Delay in a *Drosophila* Model of NGLY1 Deficiency

**DOI:** 10.1534/g3.118.300578

**Published:** 2018-05-07

**Authors:** Tamy Portillo Rodriguez, Joshua D. Mast, Tom Hartl, Tom Lee, Peter Sand, Ethan O. Perlstein

**Affiliations:** Perlara PBC, 6000 Shoreline Court, Suite 204, South San Francisco, California 94080

**Keywords:** N-glycanse 1, NGLY1, Pngl, *Drosophila*, disease model

## Abstract

N-glycanase 1 (NGLY1) Deficiency is a rare monogenic multi-system disorder first described in 2014. *NGLY1* is evolutionarily conserved in model organisms. Here we conducted a natural history study and chemical-modifier screen on the *Drosophila melanogaster NGLY1* homolog, *Pngl*. We generated a new fly model of NGLY1 Deficiency, engineered with a nonsense mutation in *Pngl* at codon 420 that results in a truncation of the C-terminal carbohydrate-binding PAW domain. Homozygous mutant animals exhibit global development delay, pupal lethality and small body size as adults. We developed a 96-well-plate, image-based, quantitative assay of *Drosophila* larval size for use in a screen of the 2,650-member Microsource Spectrum compound library of FDA approved drugs, bioactive tool compounds, and natural products. We found that the cholesterol-derived ecdysteroid molting hormone 20-hydroxyecdysone (20E) partially rescued the global developmental delay in mutant homozygotes. Targeted expression of a human NGLY1 transgene to tissues involved in ecdysteroidogenesis, *e.g.*, prothoracic gland, also partially rescues global developmental delay in mutant homozygotes. Finally, the proteasome inhibitor bortezomib is a potent enhancer of global developmental delay in our fly model, evidence of a defective proteasome “bounce-back” response that is also observed in nematode and cellular models of NGLY1 Deficiency. Together, these results demonstrate the therapeutic relevance of a new fly model of NGLY1 Deficiency for drug discovery and gene modifier screens.

Recessive loss-of-function mutations in the evolutionarily conserved gene *NGLY1* result in an ultra-rare genetic disease called NGLY1 Deficiency, which is characterized by global developmental delay, seizures, small head and extremities, chronic constipation, lack of tears, and floppy body (Enns *et al.* 2014). *NGLY1*, short for *N-glycanase 1*, encodes a deglycosylating enzyme that hydrolyzes N-linked glycans from asparagine residues of glycoproteins, liberating oligosaccharides for degradation and recycling (Suzuki *et al.* 2016). A comprehensive clinical snapshot by National Institutes of Health (NIH) established potential measurable clinical endpoints and a baseline of disease progression in a cohort of 12 patients (Lam *et al.* 2017).

The pathophysiology of NGLY1 Deficiency has not yet been fully resolved. Before 2014, little was known about NGLY1 function in animal development. Its elucidation is the focus of numerous research groups employing a diversity of disease models, including model organisms and human cells. Two models of the pathogenesis of NGLY1 Deficiency have been proposed.

The first model is rooted in biochemistry and defects in cellular glycoprotein homeostasis (Huang *et al.* 2015). NGLY1 is an essential component of the conserved protein quality control system known as endoplasmic-reticulum-associated degradation (ERAD), bridging p97/VCP-mediated retrotranslocation of proteins from the ER to the cytoplasm for bulk deglycosylation and subsequent degradation by the ubiquitin-proteasome system (UPS) (Suzuki 2015). The absence of cytoplasmic N-glycanase activity has been proposed to result in inappropriate cleavage of N-glycans from glycoproteins by the downstream cytoplasmic enzyme endo-β-*N*-acetylglucosaminidase (ENGase), whose normal substrate is soluble oligosaccharide liberated by NGLY1. Such glycoproteins misprocessed by ENGase would retain a single GlcNAc residue that may destabilize proteins and promote their aggregation. Suzuki and colleagues observed evidence of *N*-GlcNAc misprocessing and accumulation *in vitro* in *NGLY1^−/−^* mouse embryonic fibroblasts (Huang *et al.* 2015; Fujihira *et al.* 2017). Based on the collective work of Suzuki and colleagues, inhibition of ENGase has been proposed as a therapeutic thesis for NGLY1 Deficiency (Bi *et al.* 2017; Fujihira *et al.* 2017). Indeed, a *NGLY1^−/−^ ENGase^−/−^* double mutant mouse is viable while a *NGLY1^−/−^* single mutant displays varying degrees of lethality depending on genetic background (Fujihira *et al.* 2017). However, a *NGLY1^−/−^ ENGase^−/−^* double mutant mouse is not healthy. Additional pathogenic mechanisms are required to explain NGLY1 Deficiency.

The second model of pathogenesis is rooted in genetics and defects in the deglycosylation of specific glycoprotein clients, including but not limited to the master regulator of the conserved 26S proteasome “bounce-back” response, NFE2L1 (the transcription factor nuclear factor erythroid 2 like 1 also known as Nrf1) (Radhakrishnan *et al.* 2010). NRF1 belongs to the ancient basic leucine zipper family of transcription factors that regulate many developmental and stress response pathways in animals (Kim *et al.* 2016). The fly *NRF1* homolog, *cap-n-collar (cnc)* increases the expression of proteasome subunit genes, as well as oxidative and redox stress response pathways (Grimberg *et al.* 2011). In an unbiased screen for genetic modifiers of the proteasome bounce-back response in nematodes, Lehrbach and Ruvkun discovered that the nematode homolog of NGLY1, PNG-1, deglycosylates the ER-membrane bound isoform of the nematode homolog of NRF1, SKN-1A. They further demonstrated that deglycosylation of SKN-1A by PNG-1 is required for SKN-1A translocation to the nucleus, and transcriptional activity (Lehrbach and Ruvkun 2016).

In a complementary study, Bertozzi and colleagues revealed that NGLY1 activity is required for NRF1 signaling in mouse embryonic fibroblasts mice in the same manner that PNG-1 activity is required for SKN-1A function in nematodes (Tomlin *et al.* 2017). In fact, they showed that chemical inhibition of NGLY1 function potentiates cytotoxicity caused by proteasome inhibition in human cancer cell lines (Tomlin *et al.* 2017), which mirrors the observation in nematodes that png-1 loss-of-function mutants are extremely hypersensitive to proteasome inhibition by bortezomib (Lehrbach and Ruvkun 2016). Jafar-Nejad and colleagues showed that the fly *NGLY1/Pngl* is required during embryonic and larval development in *Drosophila* for post-translational processing of *Dpp*, the fly homolog of the conserved bone morphogen protein (BMP) family (Galeone *et al.* 2017), opening up the possibility that NGLY1 is required for the function of multiple glycoprotein clients.

Here we carried out phenotyping, high-throughput assay development and a chemical-modifier screen on a new fly model of NGLY1 Deficiency, herein referred to as *Pngl^PL^*. Of ∼2,650 bioactive compounds, the ecdysteroid insect molting hormone 20-hydroxyecdysone (20E) partially suppressed global developmental delay in mutant homozygotes. Expression of a human *NGLY1* transgene in the prothoracic gland (PG) and sites of ecdysteroidogenesis partially rescued global developmental delay in mutant homozygotes. These data indicate that defects in ecdysone-producing tissues contribute to the global developmental delay of the *Pngl^PL^* flies. *Pngl^PL^* flies are also hypersensitive to the proteasome inhibitor bortezomib and the organic solvent dimethyl sulfoxide (DMSO). Together, these observations combined with other results in the literature (Owings *et al.* 2018) can be accommodated by a model wherein *NGLY1*/*pngl* is required for *NRF1*/*cnc* function in the *Drosophila* neuroendocrine axis.

## Methods

### Pngl allele generation

**Figure 1:**
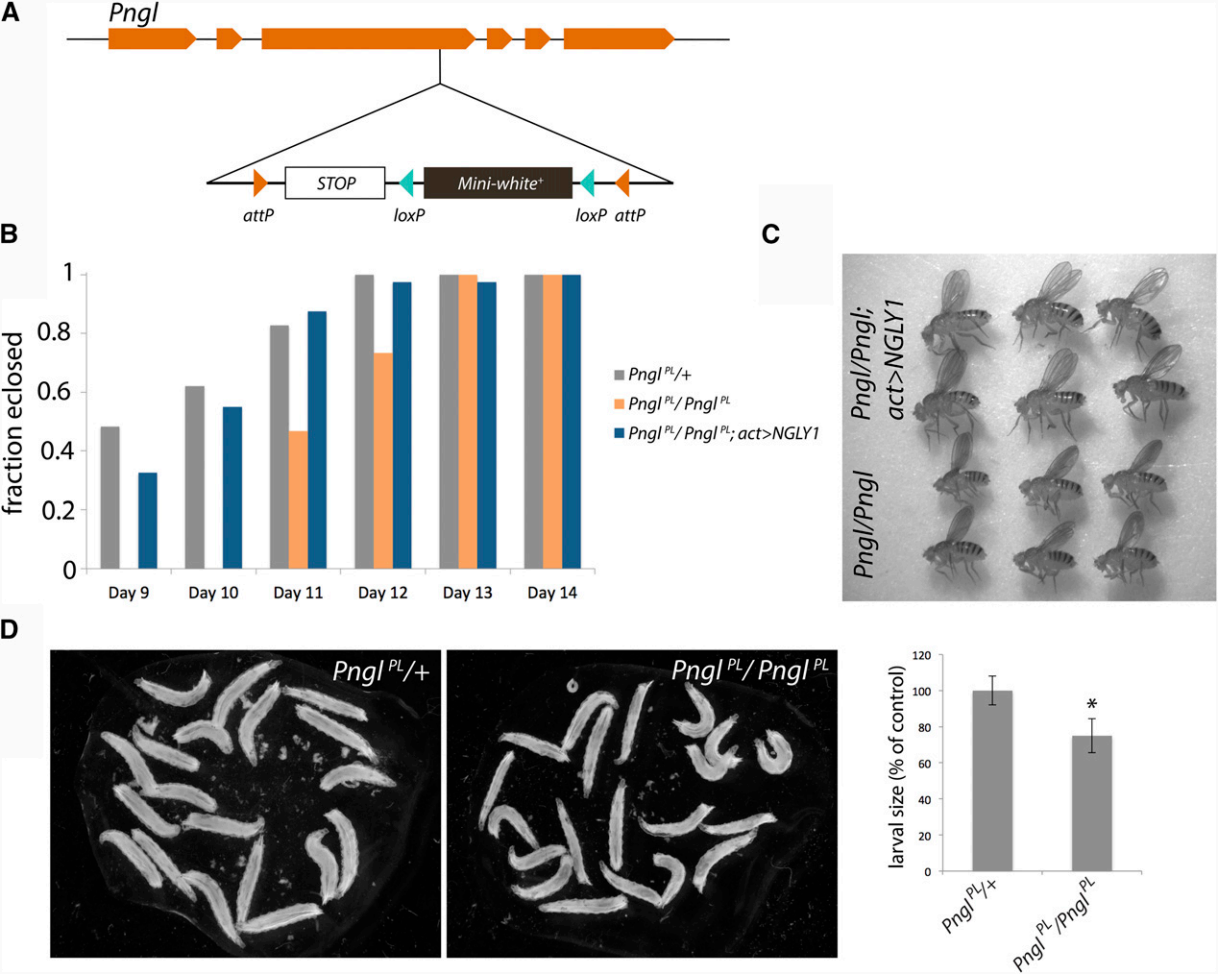
Genotyping and phenotyping *Pngl^PL^* allele. A) We used CRISPR to create an allele of *Pngl* with a stop codon and white + transgene inserted upstream of the PAW domain. Sequencing confirmed the integration of the stop codon and *mw+* immediately 3′ to bp 1906699 (Release 5.57) in chromosome 2R (NT_033778.3). B) The fraction of *Pngl^PL/+^* heterozygote, *Pngl^PL/PL^* homozygote, or *Pngl^PL/PL^;Actin > NGLY1* flies that eclose over time normalized to the total surviving flies for each genotype. Flies were grown on standard untreated media. The ratio of flies emerging in this experiment for *Pngl^PL/PL^*, *Pngl^PL/PL^* ; *Act > hNGLY1*, and *Pngl^PL^/CyO is* 15:40:137 individuals. Ubiquitous expression of a human *NGLY1* transgene rescued the 2-day development delay to eclosion observed in *Pngl^PL^* homozygotes. C) *Pngl^PL^* homozygous adult flies are smaller than homozygous siblings expressing the human *NGLY1* transgene. D) *Pngl^PL^* homozygotes are 25% smaller than their heterozygous siblings (*P* < 0.01; Student’s *t*-test).

A cassette containing a stop codon and *mw+* was cloned into a modified version of pUC57. Homology arms to the *ngly1* locus were cloned upstream and downstream of the cassette. The guide RNA (GCTGAGGAATAACTTTCGAT CGG) was cloned into pCDF3 (Port *et al.* 2014). pCDF3 and pUC57 with *Pngl* homology arms, stop codon, and *mw+* were injected into *vas-Cas9* (Bloomington stock #51323). Two independent *mw+* F1 strains were established and balanced stocks were created. Sequencing confirmed the integration of the stop codon and *mw+* (Figure 1A) immediately 3′ to bp 1906699 (Release 5.57) in chromosome 2R (NT_033778.3).

### Rate of eclosion and rescue by human ngly1 transgene studies

*w*, *Pngl^PL^/CyO*; *actin-switch-GAL4* males were crossed to *Pngl^PL^/CyO,Act-GFP*; *UAS-human-NGLY1* virgin females. The phenotypes of eclosing flies were recorded on days 9-14 after parents were mated. We found that the *actin-switch-gal4* transgenic strain expressed *GAL4*, despite the absence of RU486.

### Fly strains

*Actin5C-switch-GAL4* (stock #9431) was obtained from the Bloomington stock center. *Pngl* excision alleles and *UAS-human-NGLY1* were obtained from Dr. Hamed Jafar-Nejad. *2_286-GAL4* driver was obtained from Dr. Kaye Suyama. *phm-GAL4* and *spok-GAL4* drivers were obtained from Dr. Michael O’Connor.

### Larval size measurements

0-4 hr old larvae were placed into petri dishes with standard fly food media for 3 days at 25°. Then, larvae were removed from the food, rinsed in PBS, and placed in PBS to be imaged. The areas of nineteen heterozygous and twenty homozygous larvae were measured in ImageJ.

### Plate preparation for chemical modifier screening

100nL compound or DMSO (vehicle) was dispensed from mother plates into wells of 96 well daughter plates using the Echo acoustic dispenser (LabCyte). Then, 100μL of molten standard fly food media (molasses, agar, yeast, propionic acid) lacking cornmeal, but carrying 0.025% bromophenol blue, was dispensed using a MultiFlo (Bio-Tek Instruments). Bromophenol blue dye in the larval gut increases their signal over background later during imaging. Plates were placed on a plate shaker and shaken for 1 min, during which, the molten agar solidified with thoroughly mixed compound/DMSO in each well. Plates were then covered with adhesive aluminum seals and stored at 4° for up to two weeks.

### Culturing fly larvae in 96-well plates

*Pngl^PL^/CyO*, *Act-GFP* flies were cultured in a large population cage (Genessee) for up to two weeks, where they laid eggs on grape juice agar trays (Genessee) coated with a thin strip of active yeast paste. Egg collections were restricted to ∼6 hr during morning hours and were placed into 25° for ∼24 hr. ∼0-6hr old 1^st^ instar larvae were rinsed off of the trays with room temperature water and collected in funnel-attached 40 micrometer sieves typically used for cell straining. To remove embryo contamination, the larvae/embryo mixture was incubated two times in 10.3% inoculation solution (sodium chloride, sucrose, 10% Triton X-100) for three minutes. Most embryos float to the top of this solution after three minutes, while larvae remain in suspension. Embryos from the mixture’s solution can therefore be removed with a 10ml pipette. Embryos were then added to sorting solution (Polyethylene glycol, COPAS 200x GP sheath reagent, Tween20) and drawn into a BioSorter (Union Biometrica) for sorting and dispensing into 96 well compound/media bearing plates. Three GFP negative homozygotes were gated from heterozygotes and dispensed into the 11 left most columns of the plate, and wells G12 and H12. Three GFP positive heterozygotes were dispensed into wells A12, B12, C12, D12, E12, and F12. Plates were then covered with permeable adhesive seals and incubated at 25° for three days. Approximately 18 plates were dispensed into per day, and larval viability was not affected by their time submerged in dispensing solution (<4 hr).

### Preparation of plates for imaging

On the terminal day of the assay (day 3), gas permeable seals were removed and plates were filled with 20% sucrose solution made acidic with hydrochloric acid (pH 2) and carrying defoamer (Five Star Defoamer 105-2 oz). Plates were then vortexed and more solution was added to suspend larvae to a fixed focal plane. Plates were then imaged.

### Image and data analysis

The Fly Imager uses a Sony a7r ii camera, controlled over USB by the gphoto software to generate full plate images that are then run through a larval detection algorithm. The algorithm first builds an image of what the well would look like when it is empty. To do this it excludes areas near edges (since those are probably larvae) and interpolates across the gaps. It then looks at the difference between the image and estimated empty background, selecting areas with a high difference to be larvae.

### 20E feeding and effect on developmental timing

20E (Enzo) was dissolved in 100% ethanol and added to molten standard fly food media at 200µM and added to vials. An equivalent set of vials with an equal amount of ethanol was added for negative controls. 0-6 hr larvae were dispensed with the BioSorter into the vials and incubated at 25° for 12 days. The number of larvae that pupariated were recorded on key days at 8 am, 12 pm, and 4 pm, and the number of adults that eclosed were recorded on days 9 – 12 at a single time.

### Rescue of developmental delay with ring and prothoracic gland expression of human NGLY1

Ring gland expression of human *NGLY1* was driven using the *GAL4/UAS* system (Brand and Perrimon 1993) and ring gland drivers *2_286-GAL4*, *spok-GAL4*, and *phm-GAL4* in *Pngl^PL/Ex20^* compound heterozygotes. Adults were allowed to lay eggs for 8-16 hr, and the genotypes for all eclosing flies were recorded. The number of ring gland rescued flies eclosed was normalized to the number of eclosed sibling homozygotes not carrying the driver.

### Bortezomib feeding and effect on developmental timing

Bortezomib (Selleck Chem) was dissolved in DMSO at 100mM stock concentration and added to molten standard fly food media to a set of 35mm petri dishes at testing concentrations while maintaining a final 0.025% DMSO concentration. An equivalent set of 35mm petri dishes with an equal amount of DMSO was added for negative controls. 0-6 hr larvae were dispensed with the biosorter into the 35mm petri dishes and incubated at 25° for 3 days. The number of larvae that were still alive were recorded, and imaged to quantify the sizes with FIJI software.

### Data Availability

The authors state that all data necessary for confirming the conclusions presented in the article are represented fully within the article. All strains are available upon request. Supplemental material available at Figshare: https://doi.org/10.25387/g3.6227189.

## Results

### Generation of the Pngl^PL^ fly and characterization of its phenotypes

We used CRISPR/Cas9 to create a new allele of *Pngl* with a premature stop codon and a selectable marker inserted upstream of the PAW domain, herein referred to simply as “*Pngl^PL^*^”^ (Figure 1A). We rationalized that this truncated allele better reflects patient alleles, as no *NGLY1* null alleles have been observed in individuals affected by NGLY1 deficiency. We benchmarked *Pngl^PL^* against the previously described *Pngl* genetic null mutant generated by *P*-element excision *(Pngl^ex20^)*, which causes developmental defects and semi-lethality with few adult escapers (Funakoshi *et al.* 2010). *Pngl^PL^* homozygotes are delayed in the larval-to-pupal transition by one day, and delayed to eclosion by 2 days when grown on standard media (Figure 1B). As three-day-old larvae, *Pngl^PL^* homozygotes are ∼75% the size of their heterozygote siblings (*P < 1X10^−10^*, *Student’s t-test*) (Figure 1D).

Consistent with semi-lethality observed in other *Pngl* mutants, *Pngl^PL^* homozygote mutants survive to pupation, but only 32% of flies emerge as adults when reared at 25° (Funakoshi *et al.* 2010, Galeone *et al.* 2017). We also found the degree of this lethality in *Pngl^PL^* homozygote mutants was temperature dependent. Specifically, the pupal lethal phenotype was cold-sensitive, and at temperatures at or below 21° no adults emerged. *Pngl^PL^* homozygotes that survive to adulthood are sterile, which is also consistent with the effects of previously characterized alleles, *Pngl^ex20^* and *Pngl^ex14^* (Funakoshi *et al.* 2010, Galeone *et al.* 2017).

Time to eclosion in *Pngl^PL^* homozygotes is completely rescued by ubiquitous expression of a human *NGLY1* (*hNGLY1)* transgene (Figure 1B). The small body size phenotype of *Pngl^PL^* homozygote adults is also rescued by ubiquitous expression of *hNGLY1* (Figure 1C). These results when flies are reared in standard vials suggested phenotypes suitable for high-throughput, whole-organism phenotypic screening at each stage of fly development from 1^st^ instar larvae onwards.

We decided to optimize a high-throughput larval size assay in 96-well plates for several reasons. Assay miniaturization from 30mL vials to 96-well plates involves reducing the number of animals tested by a factor of 10, *e.g.*, 30 animals per vial *vs.* 3 animals per well. The larval size difference between *Pngl^PL^* heterozygote larvae *vs.* *Pngl^PL^* homozygote larvae was more significant in 96-well plates than the timing to pupation difference or the timing to eclosion difference. A 3-day assay *vs.* a 7-11 day assay allowed for faster optimization cycle times. Time to pupation and time to eclosion would be secondary assays in vial format to ensure primary screening hits rescue global developmental delay, not just developmental delay in larvae.

**Figure 2:**
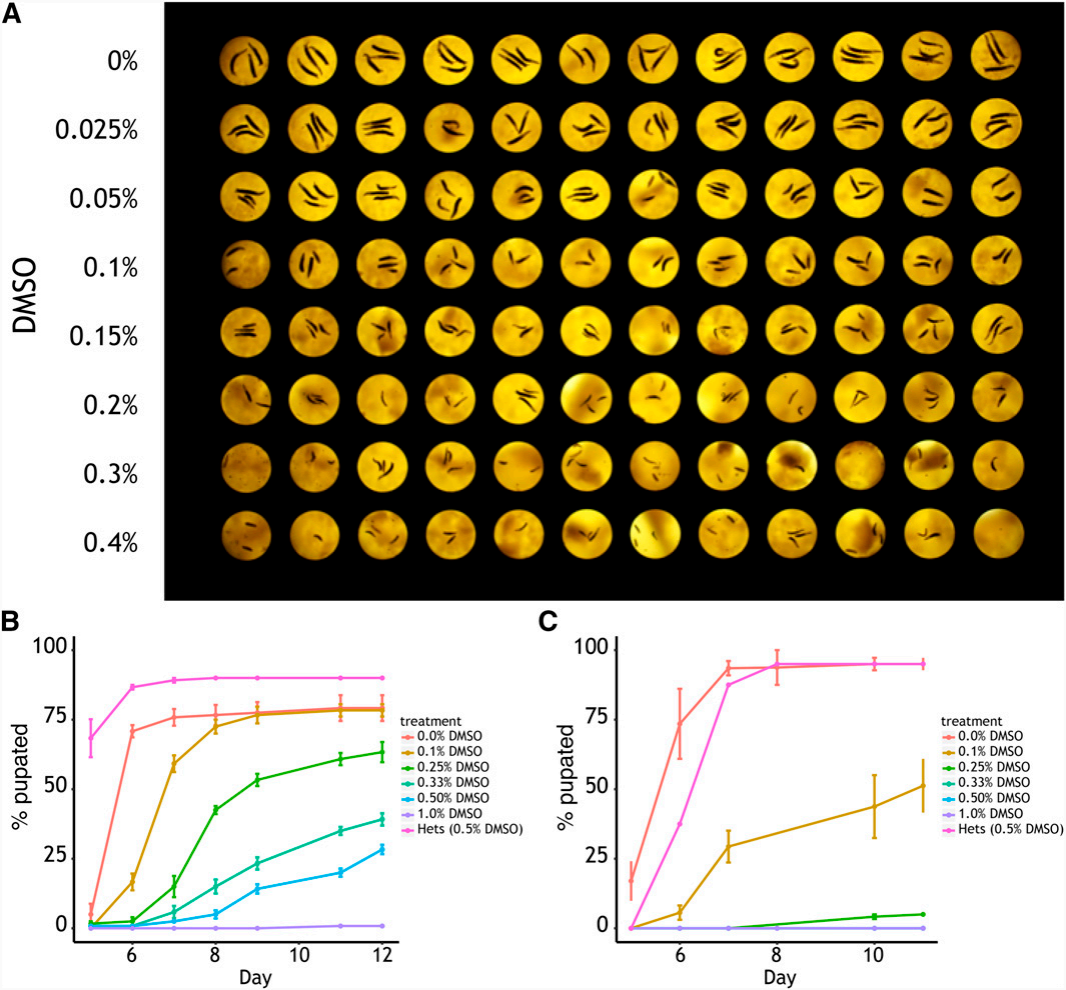
*Pngl^PL^* homozygotes are hypersensitive to dimethyl sulfoxide (DMSO). A) An image of *Pngl^PL^* homozygous larvae grown in 96-well plate format on food dosed with different concentrations (0–0.4%) of DMSO. *Pngl^PL^* homozygous larvae are hypersensitive to DMSO, a decrease in larval size is observed starting at 0.025% DMSO and larval size is decrease as the dose of DMSO is increased. The time to pupation of B) *Pngl^Ex20^* (n = 3 replicates, 20-30 individuals/replicate) or C) *Pngl^PL^* (n = 5 replicates, 20-30 individuals/replicate) homozygous larvae reared on food treated with DMSO. Homozygous mutant larvae exhibit hypersensitivity to DMSO showing a delayed time to pupariate and increased lethality.

As a prelude to drug screening, we established the tolerability of *Pngl^PL^* homozygote larvae to dimethyl sulfoxide (DMSO), the organic solvent for almost every compound library, including the Microsource Spectrum collection. The maximum tolerated dose of DMSO, therefore, sets a ceiling on the final assay screening concentration. Unexpectedly, we observed that *Pngl^PL^* homozygotes are extremely hypersensitive to DMSO compared to the heterozygote control (Figure 2). We estimated a maximum tolerated dose in the *Pngl^PL^* homozygote of 0.1% v/v DMSO (14mM) (Figure 2A, C). Surprisingly, larvae homozygous for *Pngl^PL^* are several fold more sensitive than those homozygous for the null allele, *Pngl^ex20^* (Figure 2B). The *Pngl^PL^* truncates the protein before the PAW domain, which binds mannose (Suzuki *et al.* 2016), but leaves the catalytic domain intact. This suggests to us that at least some of the DMSO sensitivity we observe is not simply due loss of Pngl activity. In comparison, wild-type *Drosophila* exhibit adult lethality starting at 1% v/v DMSO, larval lethality ranging from 2% v/v to 3% v/v DMSO, and a “no observed adverse effect level” (NOAEL) dose of 0.3% v/v DMSO (Figure S1; Nazir 2003). DMSO could be acting as a general stressor, or possibly inducing oxidoreductive stress, specifically. For the purposes of drug screening, we exploited DMSO as a sensitizer in the larval size assay. Exposure of mutant larvae to 0.1% v/v DMSO, which would entail a final assay screening concentration of 10µM for each library compound, was potent enough to increase the dynamic range of the assay while sparing larval lethality.

### A high-throughput image-based larval size chemical modifier pilot screen yielded one validated hit, 20-hydroxyecdysone (20E)

We posited that the small larval size phenotype of *Pngl^PL^* homozygote mutants could be used to discover small-molecule suppressors that provide insight into the pathophysiology of NGLY1/*Pngl* deficiency in the fly. To that end, we developed a novel image-based assay to culture *Drosophila* larvae in clear-bottom 96-well plates where each well either contains a unique small molecule or vehicle. Using a 96-well-plate format allowed us to take advantage of existing lab automation for managing multi-well plates in high-throughput screening (HTS) applications, including a whole-organism sorter and dispenser. Our method includes steps to dissolve and dilute pre-existing fly food so that larvae can be floated to a fixed focal plane and then imaged with custom plate imager. Software was written to measure the overall area of floated larvae to enable the identification of small molecules that significantly increase the size of *Pngl^PL^* homozygote larvae *vs.* vehicle-fed larvae.

**Figure 3:**
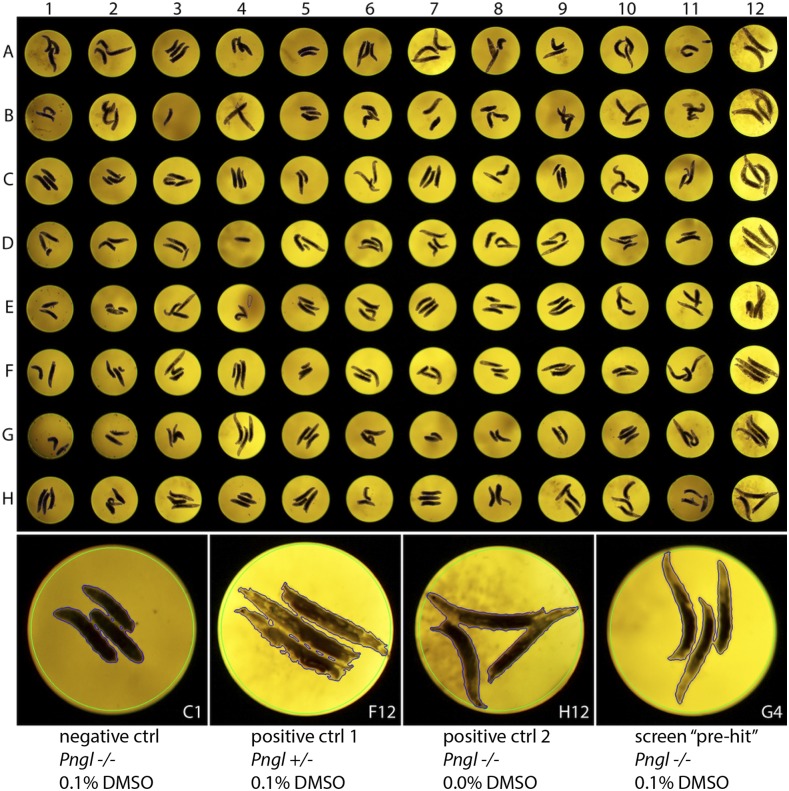
Layout of an *Pngl^PL^* assay plate with examples of controls and pre-hit compound. Larvae were cultured in 96-well plates with negative controls on the left most wells (column 1) which carried *Pngl^PL^* homozygous with vehicle DMSO at 0.1%. 80 testing wells were in the middle (columns 2-11) which carried *Pngl^PL^* homozygous larvae with a compound at 1μM, and DMSO at 0.1%. Right most top wells (12A-12F) contain positive control group 1 with *Pngl^PL^* heterozygous larvae and 0.1% DMSO. Right most bottom wells (12G-12H) contain positive control group 2 with *Pngl^PL^* homozygous larvae without DMSO. The compound in well G4 was considered a pre-hit compound.

We selected the Microsource Spectrum collection for a pilot screen. The library contains 2,532 unique compounds including ∼600 FDA approved drugs, ∼800 compounds that have reached clinical trial stages in the USA, ∼400 drugs that have been marketed in Europe or Japan but not the USA, ∼600 bioactive tool compounds, and ∼800 natural products. Three larvae per well were cultured in 96-well plates with control wells comprising the two outermost columns. We used *Pngl^PL^* homozygous larvae fed vehicle (0.1% v/v DMSO) as a negative control. We include two positive control groups: the first group consist of *Pngl^PL/+^* heterozygous larvae fed vehicle, and the second group consists of *Pngl^PL^* homozygous larvae cultured without DMSO. The remaining 80 wells contained *Pngl^PL^* homozygous larvae fed a unique library compound at 10µM plus 0.1% v/v DMSO. An image of a representative drug screening plate with zoomed-in reference wells is shown in Figure 3.

**Figure 4:**
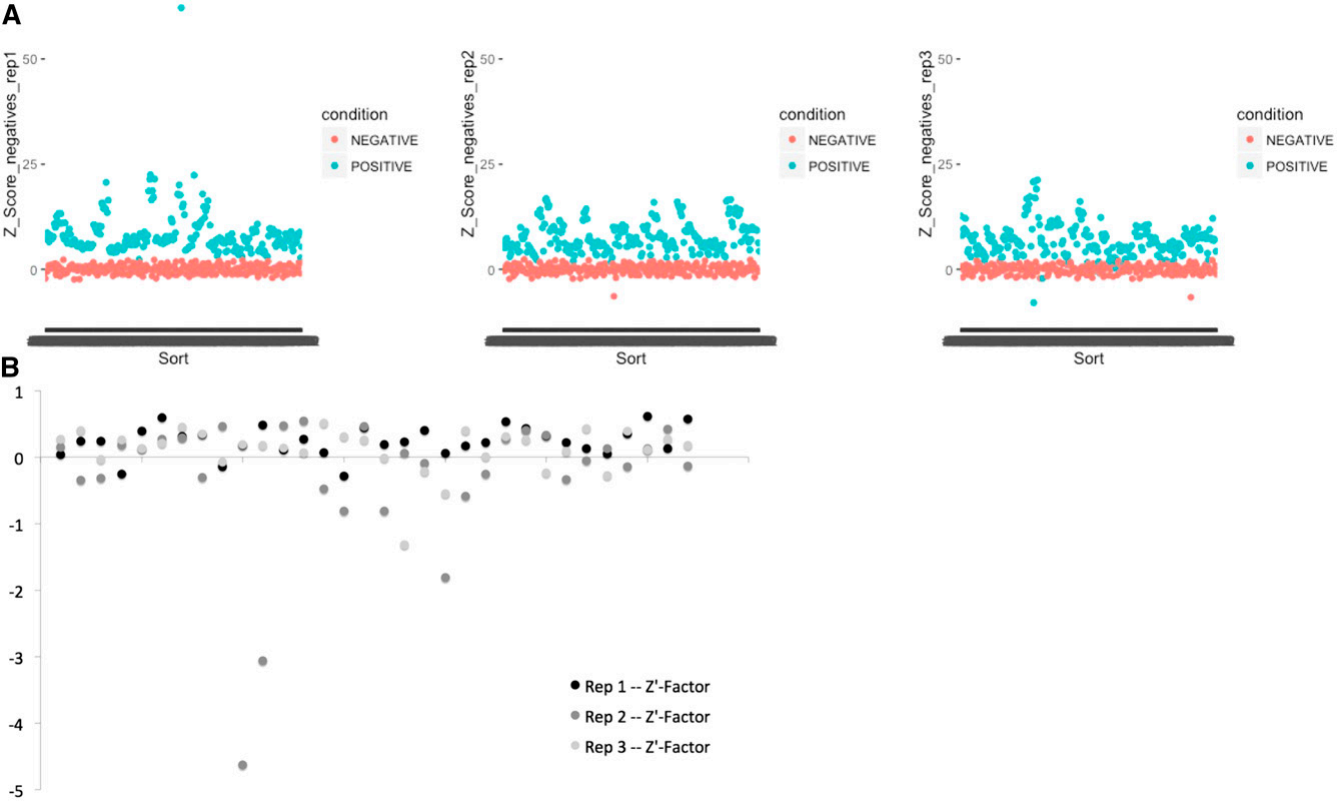
The assay had a consistent difference between positive and negative controls. A) A consistent separation between positive and negative controls with Z’Factor >0, indicated an assay that could significantly identify chemical suppressors. B) Three replicates of a 32 plate library were analyzed and 73 of 96 plates had a Z’Factor >0.

**Figure 5:**
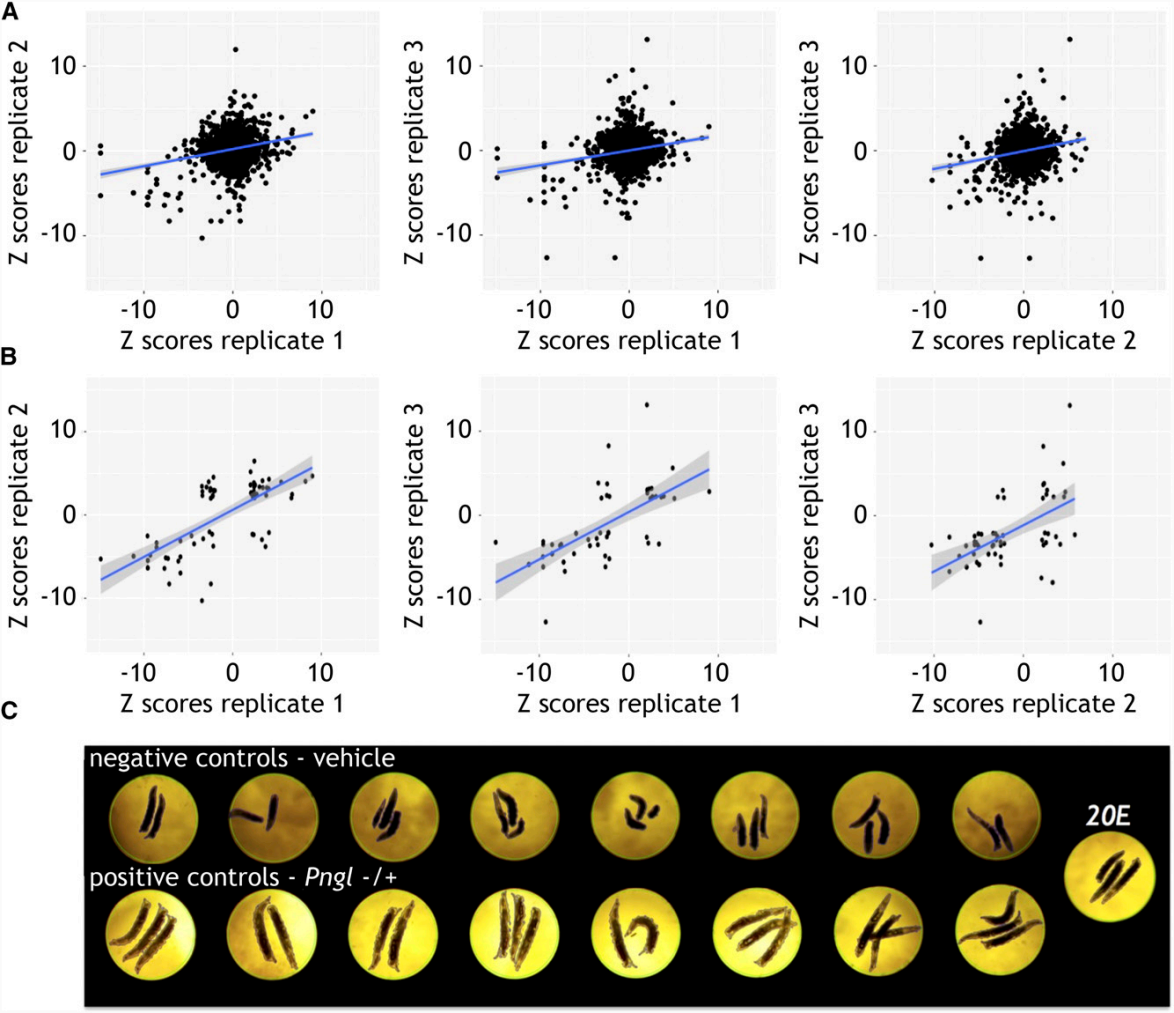
Positive correlation between 3X replicates. A) The three pairwise comparisons of Z-scores show positive correlations indicating that a set of small molecules could modify the small larval size phenotype B) The positive correlations between replicates is more evident when only plotting Z-scores of < -2 or > 2. C) Larval size is partially rescued when 20E is fed to *Pngl^PL^* homozygous larvae.

We performed the screen in triplicate, meaning three independent biological replicates. There was statistically significant separation between positive and negative controls (Figure 4A). On average, *Pngl^PL^* homozygotes were half the size of *Pngl^PL/+^* heterozygotes, although some variation in size was observed in each genotype. 75% of all screening plates (73 of 96) had a Z’ factor > 0; most of the screening plates with high variability belonged to the second replicate (Figure 4B). A weak positive correlation existed in pairwise comparisons of each biological replicate when all data points are included (Figure 5A). When we only included wells with Z scores less than -2 or greater than 2, the positive correlation increased significantly on average (Figure 5B). For example, in the pairwise comparison of replicate 1 *vs.* replicate 2, the correlation improves from R^2^ = 0.05977 in the full dataset to R^2^ = 0.48171 in the reduced dataset (with positive and negative controls removed as well).

**Figure 6:**
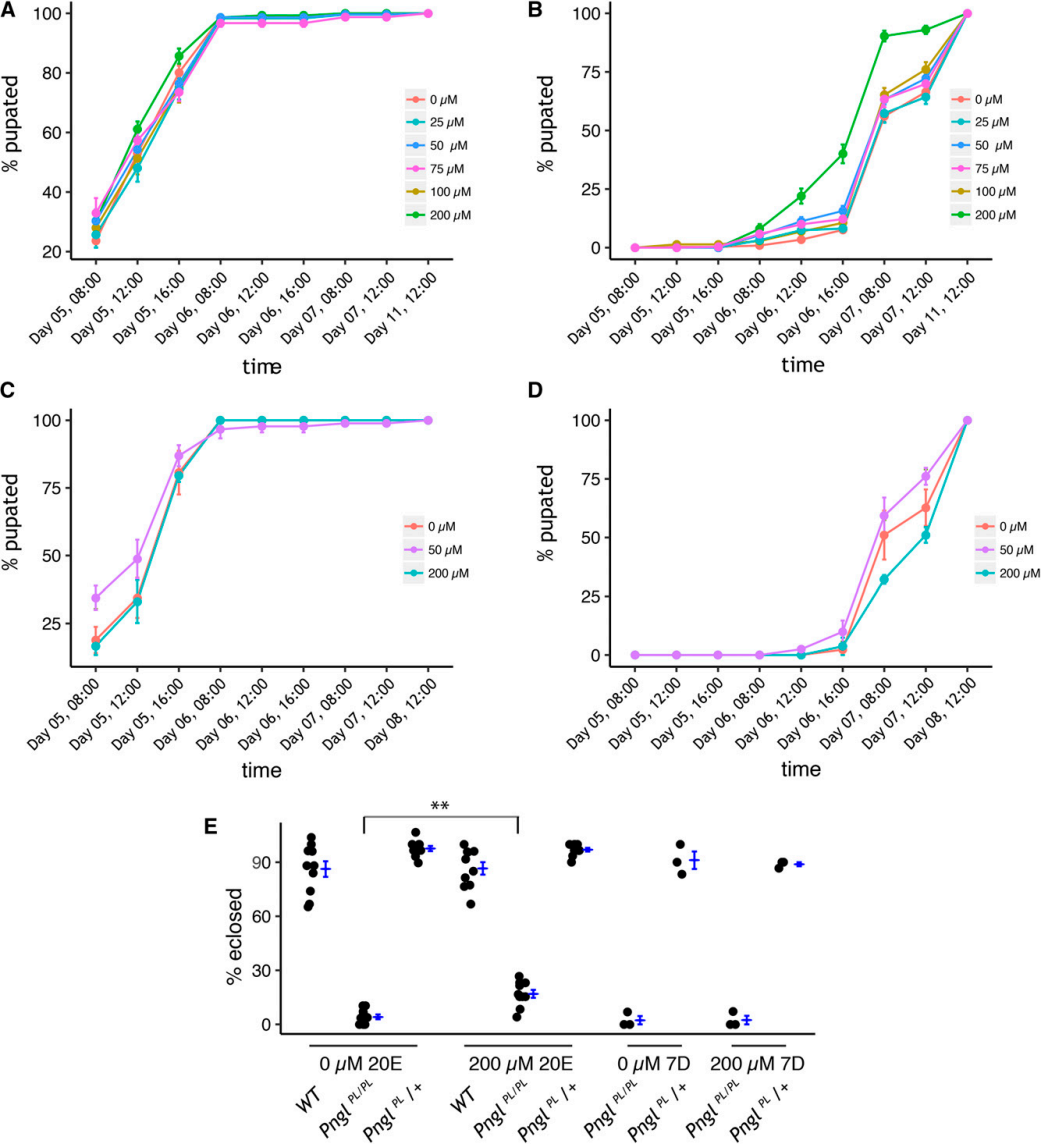
20-hydroxyecdysone (20E), but not an earlier ecdysone pathway precursor (7-dehydrocholesterol), partially rescues developmental delay and lethality in *Pngl^PL^ mutants*. The time to pupation of A) *Pngl^PL/+^* heterozygous or B) *Pngl^PL^* homozygous larvae reared on food treated with different concentration of 20E (n = 10 replicates, 20-30 individuals/replicate). 20E did not impact developmental rate of heterozygous *Pngl^PL^* larvae to pupation, but partially rescued development delay of homozygous *Pngl^PL^* larvae to pupation at 200µM. The time to pupation of C) *Pngl^PL/+^* heterozygous or D) *Pngl^PL^* homozygous larvae reared on food treated with different concentration of 7-d (n = 3 replicates, 25-30 individuals/replicate). 7-d did not impact developmental rate of heterozygous or homozygous *Pngl^PL^* larvae to pupation. E) The fraction of animals surviving to eclosion. 20E, but not 7-d, rescued larval lethality of *Pngl^PL^* homozygous at 200µM. (n = 10 replicates, 20-30 individuals/replicate; n = 3 replicates, 25-30 individuals/replicate, respectively).

We initially considered 162 pre-hits with a Z score of > 2 in two of three biological replicates as primary screening positives (Table 1). Over two-thirds of those pre-hits proved to be false positives for one of two of the following reasons. First, uneaten fly food in the well occasionally increased the image background artificially inflating the calculated area of the larvae. Second, because *Pngl* mutants are hypersensitive to DMSO, any failure in compound dispensing or variability in DMSO levels due to hydration resulted in larger larvae. Forty-five compounds with a Z-score of > 2 in two of three replicates were considered further because their wells did not have obvious high background or low/no DMSO. The raw images of the wells containing those 45 compounds were manually inspected, and 18 appeared to have larvae larger than the negative controls in the same plate. Ultimately, these 18 unique compounds were found to have a Z of > 2 in at least two of three replicates. All 18 of the pre-hits from the screen were ordered, dissolved, and retested in attempt to reproduce rescue in vial format with larger numbers of animals (Table 2). One compound, 20-hydroxyecdysone (20E), partially rescued the developmental delay of *Pngl^PL^* homozygote larvae development to pupae (Figure 6B), but had no effect on developmental timing of *Pngl^PL^* heterozygote larvae (Figure 6A). Moreover, the suppressive effect of 20E persisted to adulthood, resulting in a statistically significant fourfold increase in eclosion percentage (Figure 6E; P<0.01 Student’s *t*-test).

**Table 1:** Whittling Screen pre-hits to a set of promising compounds to test in validation studies

	Compounds with Z > 2 in 2 of 3 replicates	High background	Low/No DMSO	Without high background or Low/No DMSO	Clear difference from controls found by manual inspection
*Replicates 1 and 3*	44	30	3	11	5
*Replicates 1 and 2*	42	12	5	25	12
*Replicates 2 and 3*	76	64	3	9	5
*Total*	162	106	11	45	22

Ultimately 22 compounds were found to have a Z of >2 in two of three replicates and upon manual inspection, the wells with those cpds appeared to have larvae larger than the negative controls. Two of those 22 compounds had a Z of >2 in three of three replicates, were compounded twice in this comparison, and thus the final set of consider further were 18 unique compounds.

**Table 2:** The 18 compounds that showed promise in the screen and were considered pre-hits. These had a Z-score of >2 in 2 of 3 replicates in the primary screen

Common Name	Replicate 1	Replicate 2	Replicate 3	Retest, Phase 1*	Retest, Phase 2 ***	Retest, Phase 3 ***	Retest, Phase 4 ****
CRUSTECDYSONE	3.36	2.24	3.03	Small study indicated that 100μM rescued developmental delay to pupation	Trend supported that small size was partially rescued	Trend supported that small size was partially rescued	Positive – conclusive evidence that larval developmental delay and pupal lethality was recued.
TETRACYCLINE HUDROCHLORIDE	2.84	1.01	2.39	Z > 2, 3X @50μM	Trend supported that small size was partially rescued	Negative	Negative
HARMINE	3.37	1.92	2.41	Z > 2 in >1 replicate	Trend supported that small size was partially rescued	Negative	Negative
SULFLURAMID	4.45	6.22	−0.01	Z > 2, 3X @ 12.5μM	Larvae appeared larger than controls, but the measured areas were not.	Negative	Not tested
URSOCHOLANIC ACID	8.61	7.51	1.85	25 and 50μM could not be tested b/c of insolubility	Larvae appeared larger than controls, but the measured areas were not.	Negative	Not tested
5a-ANDROSTANE	6.46	−1.10	2.44	Z > 2 in >1 replicate	Negative	Not tested	Not tested
IRIGENIN TRIMETHYLETHER	0.73	5.62	4.90	Z > 2 in >1 replicate	Negative	Not tested	Not tested
PATULIN	−0.43	2.22	2.63	Z > 2 in >1 replicate	Negative	Not tested	Not tested
ANTIMONY POTASSIUM TARTRATE TRIHYDRATE	2.30	0.57	2.66	Z > 2 in >1 replicate	Negative	Not tested	Not tested
ALOGLIPTIN BENZOATE	2.23	8.26	−2.26	Z > 2 in >1 replicate	Negative	Not tested	Not tested
CEFEPIME HYDROCHLORIDE	3.25	−0.12	3.85	Z > 2, 2X, @50μM	Negative	Not tested	Not tested
PALMIDROL (5mM)	3.95	1.01	5.46	10μM did not rescue developmental delay or pupal lethality at 21°C.	Not tested	Not tested	Not tested
THIOGUANINE, TIOGUANINE**	3.51	−0.91	0.80	Z not >2 in > 1 replicate	Not tested	Not tested	Not tested
THIOGUANINE, TIOGUANINE	0.91	−0.02	4.97	Z not >2 in > 1 replicate	Not tested	Not tested	Not tested
CAPTAN	4.11	−3.41	3.36	Z not >2 in > 1 replicate	Not tested	Not tested	Not tested
AGARIC ACID	3.16	1.90	4.05	Z not >2 in > 1 replicate	Not tested	Not tested	Not tested
ZONISAMIDE	0.42	3.19	2.55	Z not >2 in > 1 replicate	Not tested	Not tested	Not tested
BROXALDINE	2.05	−1.05	6.62	Z not >2 in > 1 replicate	Not tested	Not tested	Not tested
PICEID	2.66	3.04	2.06	Not tested	Not tested	Not tested	Not tested

* Phase 1 was 3 or 6 reps of a dose response (3.125, 6.25, 12.5, 25, and 50μM) in the primary screen/96 well format and/or tests for rescue of developmental delay (CRUSTECDYSONE and PALMIDROL).

** The drug library had thioguanine in 2 different wells. *** Phase 2 and 3 tested whether promising pre-hits could rescue larval size at 10μM or 50μM in petri-dish experiments. ****Phase 4 tested whether promising pre-hits could rescue larval developmental delay and pupal lethality at 22.6° C.

As a control to rule out a simple ecdysone biosynthesis defect, we showed that the 20E precursor 7-hydroxycholesterol (7D) failed to rescue *Pngl^PL^* homozygote larvae development to pupae (Figure 6D). If synthesis of 20E is faulty, it is likely at a point downstream of 7D. We could not reproducibly validate any of the other 17 pre-hits, so we focused our efforts on understanding the mechanism-of-action (MoA) of 20E. Therefore, this pilot screen had an extremely low hit rate of 0.04% (1/2532).

### 20E implicates the fly neuroendocrine axis as particularly sensitive to loss of NGLY1/Pngl function

20E drives metamorphosis in *Drosophila* and arthropods generally (Faunes and Larraín 2016). Dietary cholesterol forms the basis of 20E, and all 20E precursors are synthesized in the prothoracic gland, an organ that comprises part of the larger ring gland. The immediate 20E precursor, ecdysone or “E”, is packaged into secretory vesicles, secreted, and distributed by the hemolymph throughout the animals. E is converted to 20E in these peripheral tissues, and initiates signaling cascades and gene expression inducing physiological, morphological, and behavioral changes with each molt, or developmental transition.

**Figure 7:**
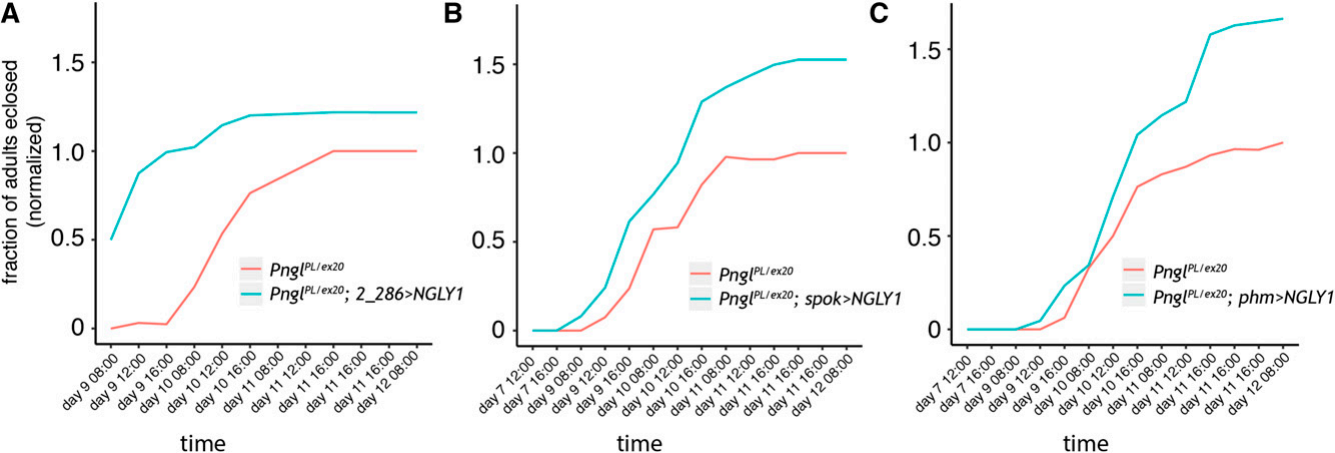
*Pngl* is necessary for normal function of the ring gland. The fraction of *Pngl^PL^ / Pngl^Ex20^* compound heterozygotes eclosed with human *NGLY1* driven by the ring gland driver (blue) A) *2_286-GAL4* B) *spookier-GAL4*, or C) *phantom-GAL4* compared to sibling controls lacking a driver (red). The reported values are normalized to the total number of eclosed *Pngl^PL^ / Pngl^Ex20^* compound heterozygotes for each experiment. A) Compound heterozygous *Pngl^PL^ / Pngl^Ex20^* larvae expressing *2_286 > NGLY1* eclosed earlier and had lower lethality than control flies (64 *Pngl^PL^ / Pngl^Ex20^*; 2_286-*GAL4* individuals compared to 120 *Pngl / CyO siblings*; 74 *Pngl^PL^ / Pngl^Ex20^*; *2_286 > hNGLY1* individuals compared to 185 *Pngl / CyO siblings)*. Compound heterozygous *Pngl^PL^ / Pngl^Ex20^* larvae expressing A) *spookier > NGLY1* or B) *phantom > NGLY1* had lower lethality at eclosion than control flies (23 *Pngl^PL^ / Pngl^Ex20^*; *spok-GAL4* individuals compared to 337 *Pngl / CyO siblings*; 43 *Pngl^PL^ / Pngl^Ex20^*; *spok > hNGLY1* individuals compared to 398 *Pngl / CyO* siblings*)*; *and* 19 *Pngl^PL^ / Pngl^Ex20^*; *phm-GAL4* individuals compared to 235 *Pngl / CyO siblings*; 63 *Pngl^PL^ / Pngl^Ex20^*; *phm > hNGLY1* individuals compared to 459 *Pngl / CyO* siblings, respectively).

To test whether the 20E insufficiency in *Pngl*-deficient mutants is autonomous to the ring gland, we expressed a *UAS*-driven *hNGLY1* transgene that can rescue global developmental delay when expressed ubiquitously (Figure 1B, C), in the ring gland with the *2-286-GAL4* driver. To control for off-target mutations due to strain background confounding our results, we tested the *trans*-heterozygous combination of *Pngl* alleles, *Pngl^PL^/Pngl^ex20^*. Most *Pngl^PL^/Pngl^ex20^* larvae not expressing the human *NGLY1* transgene eclosed on Day 10. In contrast, most *Pngl^PL^/Pngl^ex20^* larvae expressing human NGLY1 in the ring gland pupated on Day 9 (Figure 7A). Aside from the ring gland, *2-286-GAL4* also drives expression in the salivary gland, fat body, and cuticle in larvae (Timmons *et al.* 1997). We observed a similar rescue effect when human *NGLY1* transgene expression is driven by two ring-gland-specific drivers, *phantom-GAL4* (*phm*) (Figure 7B) and *spookier-GAL4* (*spok*) (Figure 7C). *phm* and *spok* encode cytochrome P450 monooxygenases in the ecdysone biosynthetic pathway (Rewitz *et al.* 2007). Driving hNGLY expression in *Pngl^PL^* homozygotes with either *phm-GAL4* or *spok-GAL4* rescued the pupal lethality phenotype (11.4% and 9.8% eclosed, respectively) compared to sibling controls (6.5% and 6.4% eclipsed, respectively). Together, these data suggest that *NGLY1*/*Pngl* is necessary for normal function of the ring gland to enable proper levels of 20E to circulate throughout the developing animal and drive developmental transitions.

### NGLY1/Pngl deficient flies are hypersensitive to proteasome inhibition

**Figure 8:**
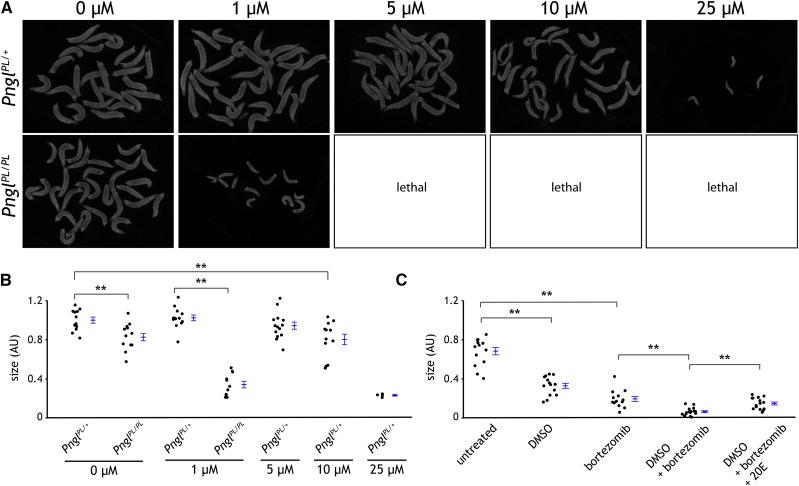
*Pngl* larvae are ≥10X more sensitive to the proteasome inhibitor bortezomib. Images A) and quantification of larval size B) of 3 day *Pngl^PL/+^* heterozygous or age matched *Pngl^PL/PL^* mutant larvae raised on food treated with 0, 1, 5, 10 or 25µM bortezomib. Black points report the size (in arbitrary units) of individual larvae. Blue lines report the mean and SE of these populations. Bortezomib delays larval developmental progression in *Pngl^PL^* homozygous larvae more severely than *Pngl^PL/+^* heterozygous larvae leading to smaller sized larvae, and is lethal for homozygotes at concentrations equal to or greater than 5 µM. For these experiments, Bortezomib was solubilized in DMSO, resulting in a final concentration of DMSO in treated food of 0.025%, which is below the threshold of effect on *Pngl^PL^* homozygous larvae. (** significant at *P* < 0.01; Student’s *t*-test) C) Quantification of larval size for *Pngl^PL^* homozygous larvae raised on untreated food, or food treated with 0.1% DMSO, 1 µM bortezomib, 0.1% DMSO and 1 µM bortezomib, or 0.1% DMSO, 1 µM bortezomib, and 133 µM 20E. 20E partially rescued the effect of bortezomib treatment on larval size in *Pngl^PL^* homozygotes. (** significant at *P* < 0.01; Student’s *t*-test).

As mentioned above, loss of NGLY1 sensitizes nematodes and human cancer cell lines to proteasome inhibition. We predicted that *Pngl^PL^* homozygote mutants would exhibit hypersensitivity to bortezomib. Indeed, bortezomib caused 100% lethality of *Pngl^PL^* homozygous larvae at 5µM, while lethality was not observed in *Pngl^PL/+^* heterozygous larvae until a dose of 25µM (Figure 8). In addition, the size of *Pngl^PL^* homozygous mutants was reduced by <50% when treated with 1µM bortezomib, and to ∼50% *Pngl^PL/+^* heterozygous larvae at ∼10µM bortezomib. These data indicate that the half-maximal inhibitory concentration (IC_50_) of bortezomib to reduce larval growth is ∼10X less in *Pngl^PL^* homozygote.

### Treatment of NGLY1/Pngl deficient flies with 20E partially rescues the effect of bortezomib

Given that both 20E and bortezomib can modify the phenotypes associated with mutations in *Pngl*, we decided to test if treatment with 20E could rescue some of the effect of bortezomib. *Pngl^PL^* homozygote mutant larvae grown on food treated with both bortezomib and 20E were significantly larger than those grown on food treated with bortezomib alone (Figure 8C).

## Discussion

We successfully generated a new *Drosophila* model of NGLY1 Deficiency, optimized a high-throughput whole-animal phenotypic assay of larval size, and then performed a proof-of-concept drug repurposing screen. While 20E itself should not be considered a drug candidate for NGLY1 Deficiency in humans, the fact that it is a chemical suppressor implicates the neuroendocrine axis in the pathophysiology of *Pngl* deficiency in flies. In other words, even though 20E is an insect-specific developmental hormone, the neuroendocrine axis and steroid-derived developmental hormones are conserved in mammals and may play a role in NGLY1 Deficiency in humans. Collectively, our findings – most strikingly, hypersensitivity of the *Pngl^PL^* mutant both to bortezomib and to DMSO – align with results observed in nematodes (Lehrbach and Ruvkun 2016) and human cells (Tomlin *et al.* 2017) that point to the essential and conserved role of NGLY1 in regulating the function of glycoprotein clients, specifically NRF1 and the proteasome bounce-back response.

In fact, we can already propose a mechanism to link NRF1 function to ecdysteroidogenesis and the neuroendocrine axis in flies. The fly homolog of NRF1 is the longest isoform of *cnc*, *CncC*, which contains a conserved N-terminal leucine rich transmembrane region targeting *CncC* to the ER (Grimberg *et al.* 2011). Specific loss of *CncC* in the prothoracic gland reduces the expression of ecdysone biosynthetic genes and results in delayed timing to pupation (Deng and Kerppola 2013). RNA interference of the Colorado potato beetle homolog of *CncC* also reduced ecdysteroidogenesis pathway gene expression and delayed timing pupation, which could be rescued by 20E supplementation (Sun *et al.* 2017). We would predict then, that as the functional equivalent of SKN-1A in nematodes and NRF1 in mammals, CncC might also be a substrate for deglycosylation by Pngl in flies. A second testable prediction is that the fly homolog of nematode DDI-1 and human *DDI2*, *rings lost* (*ringo*), acts downstream of *Pngl* to proteolyze *CncC*, generating a mature, nuclear-active species.

There are other potential explanations for how 20E might partially rescue global developmental delay in the *Pngl^PL^* mutant that do not directly involve *NRF1*/*CncC*, or that may contribute alongside loss of *NRF1*/*CncC* activity. For example, Decapentaplegic (Dpp) signaling is impaired in the ventral mesoderm of *Pngl* mutant larvae, leading to developmental defects that contribute to the lethality observed in these mutants (Galeone *et al.* 2017). Aside from local signaling, Dpp also regulates developmental timing. Dpp acts in the prothoracic gland to suppress ecdysone release by repressing the expression of genes required for ecdysteroidogenesis (Setiawan *et al.* 2017). It is possible defects in Dpp signaling in the prothoracic gland of *Pngl* mutants could cause the misregulation of ecdysone production.

Alternatively, *Pngl^PL^* mutants may not package ecdysone into secretory vesicles properly, or may be defective in secreting ecdysone. Second, signal transduction mediated by the interaction between 20E and the ecdysone receptor (EcR) might not be fully operational, and so extra 20E boosts this flawed signaling. Third, ecdysone secretion by the ring gland is induced by two neurons that synapse onto the prothoracic gland and secrete the neuropeptide prothoracicotropic hormone, or PTTH. PTTH contacts the receptor tyrosine kinase *Torso* to initiate signaling leading to ecdysone secretion. Perhaps there is a flaw in PTTH secretion or *Torso* signaling. Fourth, damage to developing larval tissues, or starvation, may impinge on 20E to fine tune organism development so that a properly proportioned and nourished animal can develop fully to adulthood. The *Pngl^PL^* mutant may have damaged tissues, for example through protein-aggregate toxicity or proteasome stress; or it may have some degree of starvation, for example if the gut cannot attain nutrients properly. 20E feeding may bypass delays induced by this hypothetical tissue damage/starvation.
