# CORRIGENDUM

**DOI:** 10.1534/g3.118.200895

**Published:** 2019-01-07

In the article by T. P. Rodriguez, J. D. Mast, T. Hartl, T. Lee, P. Sand, and E. O. Perlstein (*G3: Genes|Genomes|Genetics* 8(7): 2193–2204) entitled “Defects in the Neuroendocrine Axis Contribute to Global Development Delay in a *Drosophila* Model of NGLY1 Deficiency,” the cited number of compounds in the authors’ compound library was incorrect in two instances. On page 2193, in the article abstract, “2650-member” was corrected to “2560-member.” On page 2194, “∼2650” was corrected to “∼2560.”

